# Effectiveness and Cost-Effectiveness of a Digital Falls Prevention Program Versus Usual Care to Improve Balance, Falls Risk, and Function in Older Adults: Protocol for the Keep-On-Keep-Up Randomized Controlled Trial

**DOI:** 10.2196/78840

**Published:** 2026-02-05

**Authors:** Emma Stanmore, Amelia Parchment, Bolanle Odebiyi, Peter Bower, Chloe French, Chunhu Shi, Saima Bashir, Saima Ahmed, Dawn Dowding, Jo Dumville, Roman Kislov, Alex Thompson, Dawn A Skelton, Margaret Clarke, Chris Todd

**Affiliations:** 1 Manchester Academic Health Science Centre, Manchester, United Kingdom; 2 NIHR Applied Research Collaboration Greater Manchester, Manchester, United Kingdom; 3 School of Health Sciences, Faculty of Biology, Medicine and Health, University of Manchester, Jean McFarlane Building, Oxford Road, Manchester, M13 9PY, United Kingdom, 0161 306 7811; 4 Faculty of Business and Law, Manchester Metropolitan University, Manchester, United Kingdom; 5 Glasgow Caledonian University, School of Health and Life Sciences, Research Centre for Health (ReaCH), Glasgow, United Kingdom

**Keywords:** aged, accidental falls/prevention, postural balance, randomized controlled trial, digital health intervention, mobile apps

## Abstract

**Background:**

Falls are the primary cause of fatal and nonfatal accidental injuries in older adults. The World Falls Prevention Guidelines recommend balance-challenging, functional exercise programs as a key strategy for falls prevention, but access, uptake, and adherence to these programs in community settings remain suboptimal. Keep-On-Keep-Up (KOKU), a digital, National Health Service–approved program, was codeveloped with older adults and therapists to provide progressive, evidence-based exercises and to raise awareness of falls prevention strategies.

**Objective:**

This trial aims to investigate the effectiveness and cost-effectiveness of the KOKU digital strength and balance program for improving balance, enhancing physical function, and reducing falls risk among community-dwelling older adults.

**Methods:**

This is a 2-arm, parallel-group randomized controlled trial. A total of 196 community-dwelling older adults 60 years and older will be randomized to either the intervention group, comprising a digital strength and balance program (KOKU) alongside standard care (strength and balance exercise advice and a falls prevention leaflet), or to a control group, receiving standard care only. Participants receiving the intervention will be asked to exercise 3 times per week following the tailored and progressive program. Randomization will take place after recruitment and baseline data collection. The trial’s primary outcome measure is balance function (Berg Balance Score) at 12 weeks post randomization. Secondary trial outcomes include lower limb strength, health care utilization and health-related quality of life, self-reported concerns about falling, self-reported physical activity, falls risk, pain, mood, fatigue, self-reported falls, and acceptability and usability of the KOKU program. Intention-to-treat analysis and a cost-effectiveness analysis will be employed for trial data analysis. Qualitative interviews and focus groups will be undertaken with around 10 care providers and 13 participants to further understand views of the intervention and trial processes.

**Results:**

This study began recruitment in July 2024 and concluded in March 2025, recruiting a total of 202 participants (102 intervention group and 100 control group). Following protocol publication, data compilation and analysis will be conducted, with results anticipated to be published in 2027.

**Conclusions:**

This trial will provide important evidence on whether a digital strength and balance program can improve balance and related outcomes in older adults compared with usual care.

## Introduction

With population aging, supporting older adults to live longer, healthier, independent lives is a major societal challenge [1]. Falls are a common problem for older adults, with risk increasing exponentially with age, leading to growing health and social care costs [2,3]. These include ambulance callouts, fractures, hospital admissions, premature admission to care homes, and increased dependency. The Office for Health Improvement and Disparities estimates that in England, the total annual cost of falls-related fragility fractures was £4.4 billion (~US $6 billion) [4]. Systematic reviews of randomized controlled trial (RCT) evidence conclude that there is good evidence that strength and balance exercises are the most effective approach to improve function and reduce falls [5,6]. Effectiveness increases if the programs are challenging, of sufficient dose, progressive, and maintained [7].

In the United Kingdom, strength and balance training programs are usually delivered in community centers by qualified professionals. These programs can be effective but may be inaccessible to those who are homebound, without transport, and unavailable in some locations [8].

A growing evidence base suggests that digital interventions may be a feasible tool for the delivery of falls prevention and exercise programs among older adults [9-11]. Moreover, digital formats can incorporate health behavior change techniques such as goal setting, progress tracking, and feedback on individual progress and can be more user-friendly and appealing, which may improve engagement and adherence [12]. In comparison with face-to-face alternatives, digital platforms are relatively inexpensive, scalable, and can be safely used in the user’s own home at their convenience [12,13].

Keep-On-Keep-Up (KOKU) is a UK National Health Service (NHS)-approved digital health intervention for falls prevention [14]. KOKU can be used independently by older adults to instruct them how to undertake progressive strength and balance exercises based on the Otago Exercise Program and the Falls Management Exercise Program (FaME) [15,16]. KOKU also has 6 health literacy games to raise awareness of home hazards and ways to improve bone health, brain health, nutrition, and hydration. KOKU’s development was guided by the Healthtech Innovation Cycle [17], the EAST Behavioral Insights Framework [18], and the Medical Research Council guidance for designing and evaluating complex interventions [19]. KOKU has been iteratively co-developed with therapists and older adults, informed by international guidelines for falls prevention [7], a meta-analysis of exercises for falls prevention [6], health behavior change techniques [20], and consensus studies with outcome and reporting recommendations for falls prevention strategies [21].

This is one of the first digital evidence-informed strength and balance programs with both progressive exercises and gamified education for older people that acts as an intervention to prevent falls and functional decline. Feedback from users and stakeholders in usability and acceptability studies in the United Kingdom [22] and the United States [9] has been positive.

In this paper, we describe an RCT to evaluate the effectiveness and cost-effectiveness of a digital exercise program, KOKU, for improving balance and function and reducing falls risk in community-dwelling older adults aged 60 years and above.

## Methods

### Public and Community Involvement and Engagement

Patient and public involvement was used to inform the design of the first iteration of the KOKU program and has continued to be important for ongoing development and to inform the design of the trial. Patient and public involvement feedback has led to the development of KOKU in both iOS and Android formats to be more digitally inclusive and informed the study methods such as how to recruit participants through care providers. The study is supported by the NIHR Applied Research Collaboration Greater Manchester (ARC-GM), which has a public and community involvement and engagement lead who has convened 2 meetings during the preparation of the study methods to ensure that a diverse group of older adults’ views were incorporated into the research processes. During the design phase of this study, the research team visited community organizations supporting social care (ie, not health service providers) to explain the study and gain an insight into the organization’s daily activities and operations to inform study processes.

### Trial Design

The 2-arm parallel-group RCT will compare the effectiveness of a digital strength and balance program (KOKU) plus standard care (strength and balance exercise advice and a falls prevention leaflet) against standard care alone. The study will test the effectiveness and cost-effectiveness of the KOKU program, and the primary outcome measure will be balance, as measured by the Berg Balance Scale [23].

This study is funded by the NHS England CareTech Program, which has a remit to identify care technology solutions and provide support to gain robust evidence that could have potential for wider rollout within the care sector. The trial opened to recruitment in July 2024 and closed in March 2025, with findings expected to be published in 2027. This protocol paper follows the SPIRIT (Standard Protocol Items: Recommendations for Interventional Trials) guidelines [24] (Checklist 1) and the TIDieR (Template for Intervention Description and Replication) checklist for the description of the intervention [25] (Checklist 2).

### Participants

Participants will be recruited from a variety of organizations and social care providers based in community settings in Greater Manchester and Lancashire areas in the Northwest of England. These include adult social care teams, housing providers, and charity organizations that support older adults to remain as independent as possible in their own homes. The community organization providers will be recruited via the Greater Manchester local authorities’ adult social care managers, who will then act as gatekeepers to recruit older adults. All eligible research participants will receive a participant information sheet and will be asked to complete an informed consent form by the research team, attesting to their voluntary participation in the study.

Participants will include community-dwelling older adults aged 60 years and over, referred via community care providers in Greater Manchester and Lancashire, and eligible to take part in the study if they have the following characteristics:

- are willing and able to give informed consent
- speak English
- are able to see and safely use the tablet-based KOKU program
- can read instructions with or without glasses, as assessed by the trained research staff
- are able to mobilize indoors without the help of another person, with or without a walking aid (self-reported)

The exclusion criteria for the older adult participants are as follows:

- those who are currently using digital technologies to exercise
- currently taking part in an exercise program or undertaking physiotherapy
- those who are unable to comprehend the study procedures
- those who have medical contraindications to exercise due to unstable health or severe conditions (eg, acute illness, severe congestive cardiac failure, uncontrolled hypertension, recent fracture or surgery; myocardial infarction or stroke in past 6 mo; severe cognitive impairment; orthopedic surgery in last 6 mo or on a waiting list to have orthopedic surgery; wheelchair users; severe auditory or visual impairment; or other uncontrolled medical conditions likely to compromise the ability to exercise)

We also aim to recruit care provider staff (n=10) and a purposive sample of the participating older adults (n=13) to participate in a qualitative interview or focus group at the end of the intervention period to provide insights and feedback on the usability of the KOKU program and the trial processes.

Recruitment, randomization, data analysis, and interpretation will be undertaken by the Applied Research Collaboration-Greater Manchester (led by PB), independent from KOKU Health.

### Randomization and Blinding

Randomization will be performed using Research Electronic Data Capture (REDCap V 14.0.31 [26]) randomization module to allocate older adult participants in a 1:1 ratio to either the intervention or control group. Random permuted blocks of various sizes will be used to produce similar group sizes and preserve allocation concealment. The allocation sequence will be computer-generated and uploaded into the REDCap system by a researcher who is not involved in enrolling or assigning participants to groups.

Responsibility for enrolling participants into the trial lies with the principal investigator and staff at the sites. Allocation concealment will be ensured, as the system will not release the allocation until the participant has been randomized by a researcher not involved in recruitment, after baseline assessments have been taken. Given the nature of the trial, researchers completing data collection and participants will not be blinded. The researcher undertaking data analyses will be blinded to the intervention and control groups to minimize bias.

### Intervention

Participants allocated to the intervention group will receive an iPad for the intervention period and will be recommended to train with KOKU for 20 to 30 minutes, 3 times per week for 12 weeks. The researcher will explain and also provide written instructions on how to use the iPad and the KOKU program, including how to switch on and charge the device. Upon first opening the KOKU program, participants are asked to complete a short onboarding questionnaire (eg, age and gender) and a walkthrough featuring written, audio, and animation instructions. The researchers will give their contact details so additional digital literacy support can be provided in person or via phone calls, if required.

The digital program includes 26 strength and balance exercises that progress from seated to standing and walking exercises. As participants complete the program, the exercises progress according to responses to questions such as “Do you feel out of breath?“ or “Could you perform the exercises easily?” Each exercise has safety information and an animated coach who demonstrates correct movements with audio and written information. The exercises include heel raises, sit-to-stands, side leg raises (abductors), side, front, and back lunges, heel-to-toe walking, sideways walking, and walking backward. If participants forget to complete their weekly exercises, they are sent an app notification after 1 week to remind them to continue.

After each exercise, participants can see how many training sessions have been completed on a progress screen embedded within the KOKU program and how many sessions remain before the 12-week intervention is completed.

The KOKU program is informed by behavior change theory and techniques such as gamification to improve access and uptake of strength and balance exercises. In addition to the exercises, there are 6 complementary health literacy games to help users learn about how to promote bone health, nutrition, hydration, and safety in the home. The bone health game, for example, asks participants to choose items from a fridge that are known to deplete or enhance calcium, vitamin D, and associated nutrients or minerals. The animated character’s X-ray bones grow or shrink, and a bone barometer increases or decreases. Evidence-based messages in lay language are presented after each selection to explain why a choice of food or drink is beneficial or not for improving bone health. The embedded health literacy games were developed with international experts and are popular with users due to the gamified elements that increase engagement and enjoyment. A user instruction video provides a short demonstration of how to download and use KOKU [27].

Participants in the intervention group will also receive home exercise leaflets based on FaME [15] and Otago Exercise Program [16], and falls prevention advice alongside the KOKU digital program.

Participants in the control group will receive the same leaflets without KOKU and will be asked to undertake the exercises listed in the leaflet 3 times a week in their leisure time.

### Outcomes

At baseline, a bespoke questionnaire will capture past medical history (including fractures), medication, comorbidities, self-reported level of vision, and demographic data (age, gender, ethnicity, and socioeconomic status). Outcome data will be collected by trained researchers from all participants at baseline, 6 weeks, and at the end of the 12-week period (Table 1).

**Table 1.** KOKU^a^ trial schedule of enrollment, interventions, and assessments.

Timepoint	Prescreening	Enrolment/allocation	Study period postallocation			
			Baseline	Week 6	Week 12	Week 24
Recruitment—research team						
Identify potential care providers for study	✓					
Familiarize care providers with research project	✓					
Recruitment—care providers						
Identify potential study participants	✓					
Patient enrollment						
Eligibility screen	✓	✓				
Informed consent		✓				
Training to use KOKU		✓				
Interview/focus group					✓	
Care provider staff enrollment						
Eligibility screen		✓				
Informed consent		✓				
Training to use KOKU		✓				
Interview/focus group					✓	
Allocation—Interventions						
KOKU			✓	✓	✓	
OEP^b^ leaflet and falls prevention leaflet			✓	✓	✓	
Assessments						
BBS^c^			✓	✓	✓	
5StS^d^			✓	✓	✓	
Health questionnaires^e^				✓	✓	
Adverse events			✓	✓	✓	
Usability/acceptability questionnaires^f^					✓	
Daily falls calendar follow-up			✓	✓	✓	✓

a KOKU: Keep-On-Keep-Up.

b OEP: Otago Exercise Programme.

c BBS: Berg Balance Scale.

d 5StS: Five Times Sit-to-Stand chair test.

e Health questionnaires include 5-item Geriatric Depression Scale, Physical Activity Scale for the Elderly, Short Falls Efficacy Scale-International, Assessment of Falls Risk tool, and EQ-5D-5L.

f Usability/acceptability questionnaires include the System Usability Scale, the Short Version of the User Experience Questionnaire, and the theoretical framework for acceptability.

The primary outcome measure is balance function, as measured by the validated Berg Balance Scale (BBS) [23]. The test involves 14 assessments of static and dynamic balance and is useful in predicting the risk of falls and indicating an individual’s ability to mobilize and carry out activities of daily living.

Secondary outcomes measured at 6 and 12 weeks will be measured by the following assessments:

1. The Five Times Sit-to-Stand (5-STS) chair test [28] will be completed to assess lower limb strength. For this test, the participant is asked to stand up and sit down as quickly as possible 5 times from a standard-height chair (43.2 cm) with their arms folded. The researcher records the time taken to complete the test.
2. Health-related quality of life will be measured using the EQ-5D-5L [29], which considers 5 dimensions: mobility, self-care, usual activities, pain or discomfort, and anxiety. Each dimension is rated as one of the following 5 options: no problems, slight problems, moderate problems, severe problems, and extreme problems.
3. Mood will be assessed using the 5-item Geriatric Depression Scale. This screening tool for depression in older adults is validated to be as effective as the 15-item Geriatric Depression Scale in a wide range of settings [30].
4. The level of physical activity will be measured using the Physical Activity Scale for the Elderly questionnaire [31]. This is a validated and easy-to-administer questionnaire designed to assess physical activity in those 65 years and older.
5. Concerns about falling will be measured using the Short Falls Efficacy Scale—International [32]. The Short Falls Efficacy Scale—International is a validated and reliable 7-item tool that measures concerns about falling in relation to a range of everyday activities and is available in a number of languages [32,33].
6. The risk of falling will be assessed using the assessment of falls risk tool. This validated measure [34] has 5 items, which include a history of any fall in the previous year, 4 or more prescribed medications, diagnosis of stroke or Parkinson disease, reported problems with balance, and inability to rise from a chair without using arms. A visual analog score for pain and fatigue will be included, as these are all strong indicators of risk of falling.
7. Secondary outcomes will also consider the usability and acceptability of KOKU. At the 12-week assessment, participants in the intervention group will complete questionnaires focusing on usability, acceptability, and motivation to use KOKU. The System Usability Scale will assess the usability of KOKU from the users’ perspective [35]. The theoretical framework for acceptability will assess perceived acceptability of the KOKU system [36]. The short version of the User Experience Questionnaire will assess the usability of the KOKU system [37].
8. Falls will be assessed using daily self-report fall calendars for 24 weeks, with follow-up phone calls from a researcher to find out more details of any falls reported and any use of health care resources. This will include details on how the fall occurred, if a visit to a general practitioner (GP), hospital, or emergency department was required, and if the fall resulted in a fracture, swelling, or other self-reported injury. Fall severity will be coded as no injury, moderate injury, or serious injury based on consultation between 2 authors (CF, ES), including a registered nurse and without discussion of group allocation. Falls and all injuries will be recorded during the 6-month period, with falls defined as “an unexpected event in which participants come to rest on the ground, floor, or other lower level” [21].
9. Health care resource use: Resource use data will be collected during follow-up to measure health service utilization related to falls. This includes ambulance attendances, accident and emergency visits, general practitioner consultations, and hospital admissions (public or private). This will be analyzed descriptively as a secondary outcome and will inform the cost-effectiveness analysis.

### Data Management and Quality Assurance

Data generated will be accessible only to the research team. All data will be collected and managed using the secure, web-based software platform REDCap [26,38], hosted at The University of Manchester. The data collection forms in REDCap will use functions that check for mandatory information, data ranges, and alerts whenever data violate specific limits [38]. Paper documents, such as written consent forms, will be stored in a locked cabinet in a secure area.

Digital copies of data will be stored securely using the University of Manchester’s Research Data Storage (RDS) service while pending, during, and following analysis. Any physical copies of the data will be stored in a locked filing cabinet within a locked office building on the university campus.

Study data and materials may be looked at by individuals from the University of Manchester, regulatory authorities, or the NHS Trust for monitoring and auditing purposes, and this may well include access to personal information. A member of the participant’s care team trained for this study may also gain access to these data where essential for the identification of an individual participant.

A unique alphanumeric identification code, and subsequently a pseudonym, will be assigned for each participant. Data entry will be checked for completeness intermittently throughout the study by a researcher not involved in data collection (CS), and a trial monitoring team, independent from the sponsor and funder, will monitor progress and data management procedures every month. Direct quotations from interviews or focus groups may be used, but these will be anonymized prior to publication and in interview transcripts. Participants will be given pseudonyms in transcripts and publications.

### Sample Size

Parameter estimates are based on a previous study of a digital intervention for adults in community settings with BBS as the primary outcome measure at 12 weeks [12]. Based on data from this previous study, we estimate the SD to be 14. Correlation between baseline and follow-up was 0.81 in the previous trial, but we will use a conservative value of 0.7. To achieve 90% power at a 5% significance level, with inflation for 13% attrition, we estimate that a sample size of 196 (98 per arm) is required to demonstrate the minimal clinically important change needed to reduce falls risk and improve functional balance of a difference in means of 5 points on the BBS at 12 weeks [39]. A minimal clinically important change of 5 points in the BBS was chosen according to baseline BBS scores of 35 to 44 observed in a previous study of older adults in the same region where the trial will be conducted [12].

### Adverse Events and Risk

The main concern regarding adverse events and/or risk is whether or not the participant can come to any harm from using the KOKU digital program. No disproportionate adverse events are expected, as all of the exercises within the KOKU digital program have been well researched with older people and mimic those of a routine physiotherapy session. Participants will be trained on how to use KOKU safely and will be requested to inform the Principal Investigator in the event of any injury, after seeking appropriate medical assistance. Any participant suspected of having an adverse reaction will be immediately withdrawn from the study, with the cause of the event thoroughly investigated. The Principal Investigator will determine seriousness and causality in conjunction with any treating medical practitioners. A serious adverse event that is deemed directly related to, or suspected to be related to, the trial intervention will be reported to the ethics committee. All trial staff in contact with participants will be responsible for recording adverse events, and the adverse event procedure will be followed.

### Statistical Analysis

All statistical analyses will be performed using Stata (StataCorp) or SPSS (version 28, IBM Corp; 2021).

Baseline demographic and clinical characteristics will be summarized by trial arm using means and SD, or median and interquartile ranges for continuous variables, as appropriate, and frequencies with percentages for categorical variables.

The primary analysis will be conducted following the intention-to-treat (ITT) principle, including all participants providing evaluable outcome data irrespective of their engagement with the KOKU program or control intervention.

BBS will be analyzed using a mixed model for repeated measures (MMRM). The model will include the fixed effects of trial arm (ie, KOKU and control), time point (ie, 6 and 12 wk), and the fixed covariate of baseline scores, as well as the interactions of all fixed terms by time point, ensuring the effects are permitted to vary for each of the 2 time points. An unstructured covariance matrix will be used, and the model will be fitted using restricted maximum likelihood (RML) estimation. The primary comparison of interest is between group differences at 12 weeks. Results will be presented as regression coefficients with 95% CIs. Continuous secondary outcomes will be analyzed using a similar framework to the primary outcome.

The rate of falls will be analyzed using a parametric model for count data (eg, Poisson or negative binomial), adjusting for baseline values. The appropriate model will be chosen depending on the distribution of this variable. The incident rate ratio will be presented with a 95% CI.

All the models will be adjusted for baseline values. Thus, to minimize data loss, simple mean imputation (across the randomized groups) of the corresponding outcome data will be used. MMRM models are likelihood-based and therefore consistent with the missing-at-random assumption. Sensitivity analyses to assess the robustness of the MAR assumption will be performed for the primary outcome.

Quantitative data will be managed using REDCap [26], a secure, web-based software platform designed to support data capture for research studies, providing (1) an intuitive interface for validated data capture; (2) audit trails for tracking data manipulation and export procedures; (3) automated export procedures for seamless data downloads to common statistical packages; and (4) procedures for data integration and interoperability with external sources.

### Economic Analysis

The primary objective of the economic analysis is to assess the incremental cost-effectiveness of the KOKU program compared with usual care. A cost-effectiveness analysis (CEA) will be conducted alongside the trial from the NHS perspective, excluding patient-incurred costs. The base-case CEA will adopt a 12-week time horizon, consistent with the trial follow-up period.

Health care resource–use data collected during the telephone follow-ups of participants who report a fall will be combined with relevant unit cost data for the financial year 2023 to 2024 to calculate the total costs incurred over the study period. Unit costs will be obtained from available UK health care reference sources. For primary care, the latest Personal Social Services Research Unit reference costs will be used, while for secondary care, costs will be sourced from the latest national reference cost schedule.

Quality-adjusted life years (QALYs) will be calculated based on EQ-5D-5L utility values using the area-under-the-curve method, assuming linear extrapolation of utility between time points. EQ-5D-5L will be scored using the appropriate tariff as recommended by the National Institute for Health and Care Excellence at the time of data collection. Multiple imputation techniques will be used to handle missing data in the main analysis, with the number of imputations determined by the extent of missing data. Regression analysis will estimate incremental costs and QALYs between the intervention and control groups, adjusting for baseline covariates including sex, age, ethnicity, marital status, socioeconomic status, and baseline utility score. Model selection (such as ordinary least squares/general linear models) will be based on goodness-of-fit statistics and diagnostic tests for model specification. Incremental net-monetary benefit will be calculated using a willingness-to-pay threshold of £20,000 (~$26,900) per QALY. Cost-effectiveness acceptability curves will be produced to show the probability of cost-effectiveness across a range of thresholds.

Scenario analyses will explore the effects of various assumptions, including variations in the implementation process, the costs associated with training, and the number of imputations. Additionally, exploratory analyses will extend the trial findings and time horizon to a longer-term time horizon (2 y), providing insights into potential long-term outcomes. Both costs and QALYs will be discounted at a rate of 3.5% per annum, in accordance with National Institute for Health and Care Excellence guidelines. Sensitivity analysis will be conducted to assess the impact of assumptions related to the duration and sustainability of the intervention’s effects.

In addition to the within-trial CEA, we will undertake an exploratory budget impact and social value assessment. The impact of the intervention will be scaled to a larger, more representative population to illustrate its potential budgetary impact and the cash/noncash releasing benefits of its implementation to commissioners and other stakeholders. Return on investment and net present social/public value (NPSV) will be calculated using the methodologies outlined in the Treasury Green Book. These approaches are designed to support strategic decision-making and will complement the CEA by providing a broader value-for-money perspective for commissioners and stakeholders.

### Qualitative Analysis

All interviews and focus groups will be digitally audio recorded and transcribed verbatim. An inductive approach will be taken to identify patterns within the data. Reflexive thematic analysis will be used to analyze the qualitative data, following the steps outlined by Braun and Clarke [40]: familiarization with the data, generating codes, constructing prototype themes, revising themes, defining themes, and producing the report. This is an iterative process that involves returning to the data several times, revising codes and themes, and discussing the themes with the research team.

Integrating data from different stakeholders and the researcher aids the triangulation process, which increases the comprehensiveness and trustworthiness of the final analysis.

### Ethical Considerations

The study will be conducted in accordance with the UK Policy Framework for Health and Social Care Research and other applicable guidance. Ethical approval has been obtained from the University of Manchester Research Ethics Committees (Ref: 2024-18620-35607) prior to commencing participant recruitment. All participants will be informed about the study and will provide written informed consent before enrollment. Participation is entirely voluntary. Participants may decline to take part or withdraw from the study at any time, without providing a reason by informing a member of the research team. All data on research participants will be pseudonymized and kept strictly confidential. It will not be possible to identify participants in any publications. The principal investigator will be responsible for preserving and maintaining all data collected during and after the study. Any deviations from the protocol will be put through the appropriate ethical amendment process. The study will be conducted in full conformance with principles of the “Declaration of Helsinki,” Good Clinical Practice, and within the laws and regulations of the United Kingdom and the European Union.

## Results

**Figure 1.**
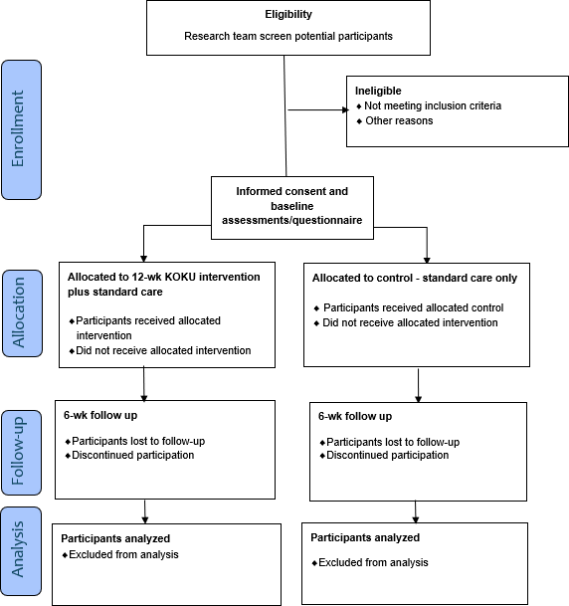
Flow diagram of study design. KOKU: Keep-On-Keep-Up.

Recruitment for this study began in July 2024 and concluded in March 2025, with a total of 202 participants enrolled. A total of 102 participants were randomized into the intervention group (n=102), and 100 were randomized into the control group (n=100). Following protocol publication, data compilation and analysis will be conducted, with results anticipated to be published in 2027. The flow of participants through the trial was recorded in compliance with the CONSORT (Consolidated Standards of Reporting Trials) statement (Figure 1).

## Discussion

### Anticipated Findings and Implications

With the growing need for self-manageable, accessible, and scalable interventions to improve function and reduce fall risk, digital health technologies present a promising option for older adults. However, evidence on their clinical effectiveness and cost-effectiveness remains limited. This trial aims to address this gap by evaluating the effectiveness of the KOKU digital program compared with usual care in improving balance, as measured by the BBS [23]. We hypothesize that participants using the KOKU digital program will show better improvements in BBS than those in the control group at 12 weeks.

In addition to balance improvements, this study will also assess other important outcomes such as concerns about falling, falls risk, and health-related quality of life (EQ-5D-5L). Concern about falling is a major factor contributing to reduced physical activity, deconditioning, and increased fall risk. Improvements in confidence may enhance overall well-being and engagement in daily activities. Furthermore, by conducting a cost-effectiveness analysis, this study will provide useful insights into whether digital interventions such as KOKU offer good value for money.

Digital programs such as KOKU may add to and enhance current service provision by offering a personalized, engaging, and home-based exercise option. If found to be effective, this program could provide an alternative or supplement to in-person programs, which may be particularly helpful for people in remote locations or with limited access to structured exercise classes.

We hope to recruit a diverse sample and have considered a variety of inequalities, including digital inequity, to reduce disparities in access, understanding, and use of KOKU. For example, KOKU was developed with older adults, including those with visual or hearing impairments as well as a range of comorbidities. In addition, we will actively recruit older adults from deprived, socially isolated communities who are digitally marginalized, and participants with impairments, comorbidities, and underserved diverse populations. To support these individuals, we can loan iPads to older adults for the duration of the study, support digital skills specifically on how to use the KOKU program, and continue to work with digital inclusion teams to extend access.

### Strengths and Limitations

This study has several strengths. Firstly, it employs an RCT design, minimizing bias and allowing for robust comparisons between the intervention and control groups. Secondly, by using the BBS as the primary outcome measure, the study employs a well-validated and clinically relevant tool to assess balance changes. Balance is widely used as a proxy for falls risk due to its being a key risk factor for falls. As a modifiable risk factor, physical interventions that target balance can directly reduce falls risk. Many falls prevention guidelines recommend balance training with improvements in balance considered a marker of intervention success [7]. Thirdly, the trial incorporates an economic evaluation, which is important for informing policymakers and health care providers about the cost-effectiveness of digital interventions, particularly those tested in real-world conditions. Additionally, by utilizing an intention-to-treat analysis, the study findings will reflect real-life effectiveness in practice.

Despite its strengths, the study has several limitations. In exercise studies, it is very difficult to blind participants or assessors, which can lead to bias. Furthermore, although unlikely, it is possible that participants in the intervention group could share information so that those in the control group have access to the intervention given that some participants were recruited from the same care providers. In addition, larger, fully powered studies are also needed to investigate the clinical and cost-effectiveness of the KOKU intervention with a primary outcome of fall rates.

### Conclusions

This trial will provide important evidence on whether a digital strength and balance program can improve balance and related outcomes in older adults compared to usual care. The findings may have important implications for promoting scalable, cost-effective digital solutions to augment current existing service provision and support healthy aging in the community.

## Supplementary material

10.2196/78840 Checklist 1 SPIRIT checklist.

10.2196/78840 Checklist 2 TIDieR checklist.

## Abbreviations

5-STS: five times sit-to-stand
ARC-GM: Applied Research Collaboration–Greater Manchester
BBS: Berg Balance Scale
CEA: cost-effectiveness analysis
CONSORT: Consolidated Standards of Reporting Trials
KOKU: Keep-On-Keep-Up
NHS: National Health Service
QALY: quality-adjusted life years
RCT: randomized controlled trial
REDCap: Research Electronic Data Capture
SPIRIT: Standard Protocol Items: Recommendations for Interventional Trials
TIDieR: Template for Intervention Description and Replication
