# Supplementary material for: Effectiveness and Cost-Effectiveness of a Digital Falls Prevention Program Versus Usual Care to Improve Balance, Falls Risk, and Function in Older Adults: Protocol for the Keep-On-Keep-Up Randomized Controlled Trial
Source: JMIR Res Protoc. 2026 Feb 5;15:e78840. doi: 10.2196/78840 (PMC12875423; doi:10.2196/78840)
Supplement: Checklist 2 [file resprot-v15-e78840-s002.pdf]

MANCHESTER 1824
The University of Manchester

A study to investigate the effects of a Digital Health Application to help improve physical function and balance in older adults

Participant Information Sheet

You are being invited to take part in a research study to find out about the effect of using a digital health program (KOKU) for older adults to help support physical function (balance and mobility). Before you decide whether to take part, it is important for you to understand why the research is being conducted and what it will involve.

Please take time to read the following information carefully before deciding whether to take part, and discuss it with others if you wish. Please ask if there is anything that is not clear or if you would like more information. Thank you for taking the time to read this.

About the research

- **Who will conduct the research?**

Dr Emma Stanmore, Dr Amelia Parchment, Ms Bolanle Odebiyi and Ms Chloe French, Division of Nursing, Midwifery, and Social Work at The University of Manchester.

- **What is the purpose of the research?**

We hope to determine the effect of using a digital falls prevention program known as, ‘KOKU’ on physical function for older adults living in the community. A total of 196 total participants will be recruited. You have been chosen because you are over the age of 60 and living in the community.

- **Am I suitable to take part?**

If you are an older adults (aged over 60 years) living in the community, you will be eligible to take part in the study. In order to take part you must be; willing and able to give informed consent; speak English; are able to see and safely use the tablet-based program and read instructions with or without glasses as assessed by the trained research staff.

- **Will the outcomes of the research be published?**

Yes, findings will be published in academic journals as well as reports to funders. All the data presented will be anonymised.

- **Who has reviewed the research project?**

This study has been reviewed and approved by The University of Manchester Research Ethics Committee [**Reference number 18620**].

- **Who is funding the research project?**

This project has been funded by NHS England.

<u>What would my involvement be?</u>

What would I be asked to do if I took part?

<table>
<tr><td colspan="2" align="center">Session 1

You will be asked to complete a consent form and some questionnaires about your age, health and a balance and mobility assessment. These assessments can take place at your own home or a convenient place such as a community centre that suits you. They are expected to take 40 minutes.</td></tr>
<tr><td colspan="2">You will be randomly allocated to either condition A or B for 12 weeks</td></tr>
<tr><td>Condition A (12 weeks)

You will be visited at home or invited to attend a training session to learn how to use the KOKU program (App). An iPad with the KOKU program will be provided for use during the study. A friend or family member is welcome to accompany you to the training session if you feel that this support would be helpful to you.

After the training, You will be requested to use KOKU for 12 weeks. This involves carrying out some simple exercises that progress from seated (e.g. heel lifts), standing (e.g. sit to stand exercise) to walking (e.g. heel to toe walking) that progress over 12 weeks. There are also health promoting games that you can</td><td>Condition B (12 weeks)

You will continue with usual care and will receive an exercise leaflet with and a falls prevention leaflet.</td></tr>
</table>

play related to home hazards nutrition, brain and bone health. During this time, a research assistant will visit you or call you once a week or as needed to provide additional support.	
Session 2<br><br>After 6 weeks, you will be asked to complete the same assessments as session 1.	
Session 3<br><br>After 12 weeks, you will be asked to complete the same assessments as session 1, plus additional questions about the KOKU program. The researcher will collect the iPad if this was provided to you during the study.	
Optional interview<br><br>A sample of up to 30 participants will be asked to take part in an interview about your experiences of using KOKU. You will be asked to complete a separate consent form. This interview will take no longer than an hour and will be audio-recorded. The interview can take place either on the telephone or in person at a convenient location that suits you such as your home or a community centre.	

- **Will I be compensated for taking part?**

No compensation will be offered for participation in this study.

- **What happens if I do not want to take part or if I change my mind?**

It is up to you to decide whether or not to take part by informing your care manager. Please feel free to discuss the study with others first before deciding whether or not to take part. If you do decide to take part you will be given this information sheet to keep and will be asked to sign a consent form. If you decide to take part you are still free to withdraw at any time without giving a reason and without detriment to yourself. However, it will not be possible to remove your data from the project once it has been anonymised, as we will not be able to identify your specific data. This does not affect your data protection rights. If you decide not to take part you do not need to do anything further.

Data Protection and Confidentiality

- **What information will you collect about me?**

In order to participate in this research project we will need to collect information that could identify you, called "personal identifiable information". We will need to collect: name, address, date of birth, gender, ethnicity, record of consent. Self-reported information about your completed exercises, mobility, fear of falling and health status will be downloaded from the provided iPad for analysis by the research team. For participants who complete the optional interview, we will also collect audio recordings of the interview. The recording will be typed out but we will remove any identifiable names or information. This transcript will be stored against an identifiable (ID) number only known by the research team. Information collected will only be used for the research purposes outlined and will be stored securely on the University of Manchester system. At the end of the project, information will be securely destroyed.

- **Under what legal basis are you collecting this information?**

We are collecting and storing this personal identifiable information in accordance with UK data protection law which protects your rights. These state that we must have a legal basis (specific reason) for collecting your data. For this study, the specific reason is that it is "a public interest task" and "a process necessary for research purposes".

- **What are my rights in relation to the information you will collect about me?**

You have a number of rights under data protection law regarding your personal information. For example, you can request a copy of the information we hold about you. If you would like to know more about your different rights or the way we use your personal information to ensure we follow the law, please consult our [Privacy Notice for Research](https://documents.manchester.ac.uk/display.aspx?DocID=37095)
https://documents.manchester.ac.uk/display.aspx?DocID=37095

- **Will my participation in the study be confidential and my personal identifiable information be protected?**

In accordance with data protection law, The University of Manchester is the Data Controller for this project. This means that we are responsible for making sure your personal information is kept secure, confidential and used only in the way you have been told it will be used.
All researchers are trained with this in mind, and your data will be looked after in the following way – the study team at The University of Manchester will have access to your personal information and they will

anonymise it as soon as possible. Your name and any other identifying information will be removed and replaced with a random ID number. The research team will have access to the key that links this ID number to your personal information. Your consent form will be retained for five years in a locked cabinet on UoM premises for audit purposes.

With your consent, we would also like to retain your contact details for five years in order to provide you with a summary of the findings for this study and also to inform you about future studies that you may be interested in. If you provide consent for this, your details will be safely stored on UoM servers in a digital folder only accessible to the study team and used only for the purposes described above.

- **Potential disclosures**

If during our conversation you reveal any information which means you may be at risk of harming yourself or others, we will be required to break confidentiality in order to put you in touch with the correct support. This may involve contacting your care manager, signposting you to relevant support services, calling a family member or friend or calling emergency services.

Please also note that individuals from the University of Manchester or regulatory authorities may need to review the study information and data collected for auditing and monitoring purposes or in the event of an incident. All individuals involved in auditing and monitoring will have a strict duty of confidentiality to you as a research participant.

What if I have a complaint?

If you have a complaint about this study that you wish to direct to members of the research team, then please contact the principal investigator:

Dr Emma Stanmore – Room 5.319, 5th Floor, Jean McFarlane Building, The University of Manchester, Oxford Road, Manchester, M13 9PL, by email emma.stanmore@manchester.ac.uk or telephone 0161 306 7645

If you wish to direct your complaint to someone independent of the research team, or if you are not satisfied with the response you have gained from the study team in the first instance, then please contact:

The Research Ethics Manager, Research Office, Christie Building, The University of Manchester, Oxford Road, Manchester, M13 9PL, by

emailing research.complaints@manchester.ac.uk or telephoning 0161 306 8089.

If you wish to contact us about your data protection rights, please email dataprotection@manchester.ac.uk or write to The Information Governance Office, Christie Building, The University of Manchester, Oxford Road, M13 9PL at the University and we will guide you through the process of exercising your rights.

You also have a right to complain to the Information Commissioner's Office about complaints relating to your personal identifiable information
Telephone 0303 123 1113
For further details, please visit: https://ico.org.uk/make-a-complaint/

Contact details

If you have any queries about the study or if you are interested in taking part then please contact the researcher:

DR EMMA STANMORE – Room 5.319, 5th Floor, Jean McFarlane Building, The University of Manchester, Oxford Road, Manchester, M13 9PL, email emma.stanmore@manchester.ac.uk or telephone 0161 306 7645.
